# Testicular necrosis and subsequent orchiectomy as a complication of inguinal mesh infection surgery: a case report

**DOI:** 10.1093/jscr/rjad074

**Published:** 2023-06-28

**Authors:** Boyodi Katanga Tchangai, Tchilabalo Matchonna Kpatcha, Komlan Adabra, David Ekoue Dosseh

**Affiliations:** Department of Visceral Surgery, Faculty of Health Sciences University of Lomé, Lomé, Togo; Department of Urology, Faculty of Health Sciences University of Kara, Kara, Togo; Department of General Surgery, Faculty of Health Sciences University of Lomé, Lomé, Togo; Department of Visceral Surgery, Faculty of Health Sciences University of Lomé, Lomé, Togo

**Keywords:** inguinal hernia, mesh, infection, orchiectomy

## Abstract

The management of inguinal hernias has been revolutionised with mesh-based techniques, which are now the gold standard. In rare cases, complications can occur, the most common being prosthesis infection. The course is unpredictable, causing considerable morbidity and multiple interventions in the case of chronicity. We treated a 38-year-old patient for an inguinal mesh infection that evolved for 8 years before definitive management. The peculiarity of this finding is the occurrence of testicular necrosis following complete removal of the prosthesis, which is likely to be related to spermatic vessel injuries. This observation shows that although healing is achieved, there may be significant sequelae, and infection prevention must be a constant concern while inserting a mesh.

## INTRODUCTION

The use of tension-free techniques has revolutionised the treatment of inguinal hernias and is currently considered the gold standard [1]. They are preferred over older techniques because of their better quality of repair and faster recovery [2]. This popularity goes along with the emergence of complications that can be considered specific to mesh-based repairs, the most feared of which is mesh infection [1, 2]. The evolution is unpredictable, and in case of chronicity, it causes considerable morbidity, multiple interventions, deterioration of quality of life and sometimes legal issues [2]. No consensus has been established on the management, particularly regarding the fate of the mesh [3]. Here, we present an unusual case of inguinal hernia mesh infection, the removal of which resulted in testicular loss. This observation illustrates the difficulties in the management of mesh infections, and the need to focus on preventive measures.

## CASE DESCRIPTION

**Figure 1:**
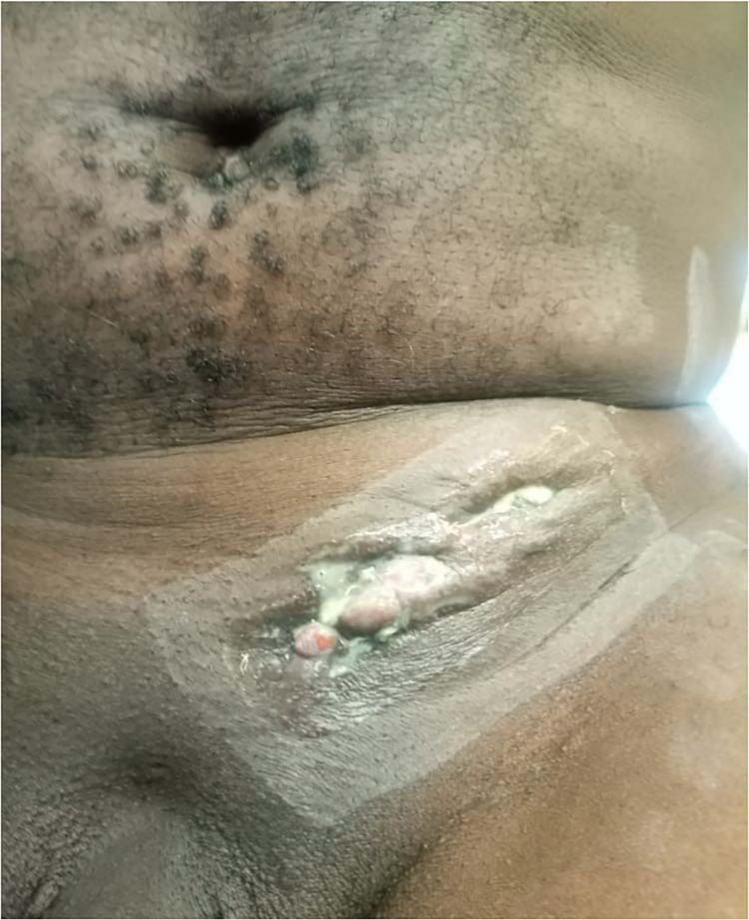
Inflammatory swelling in the inguinal area with numerous fistulous orifices discharging pus.

**Figure 2:**
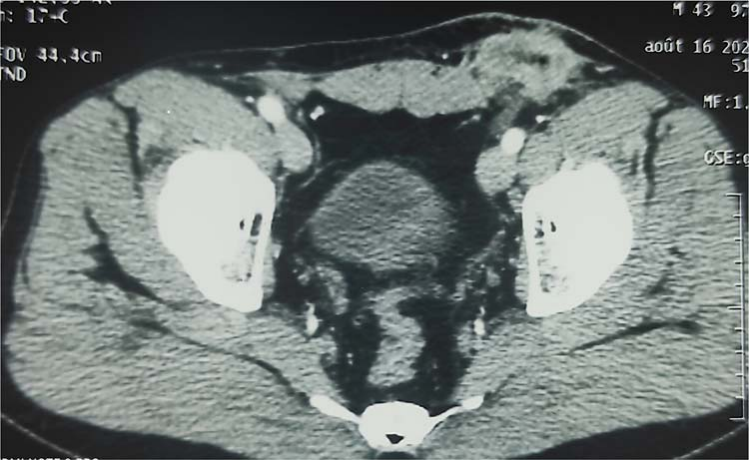
Enhanced CT scan showing well-limited mass in the left inguinal region with heterogeneous enhancement and central hypodensity related to the infected mesh.

**Figure 3:**
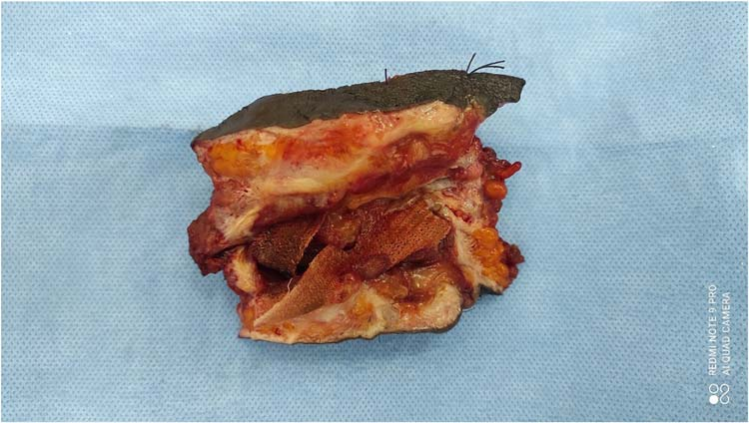
Surgical specimen showing inflammatory granuloma and sinus encircling infected mesh.

A 38-year-old patient visited our department with a purulent discharge from an inflammatory mass in the left inguinal region. He had a history of mesh repair for a left inguinal hernia performed in another hospital eight years before his admission to our hospital. Hernia repair was performed as part of a humanitarian surgical outreach. Patient experienced pain and reported purulent discharge from the surgical wound, two months after the operation. This complication was treated with local care and antibiotic therapy, the nature of which is not specified. The infection persisted despite the treatment. A second operation performed two years after the first one, allowed partial removal of the infected mesh and temporary remission of the symptoms. Apart from the surgical history, the patient had no known pathological history and was neither a diabetic nor a smoker. On admission to our department, his general condition was preserved, the body mass index was 25 kg/m^2^ and body temperature was 37.5°C. On examination of the left inguinal region, an inflammatory and painful mass was noted with several fistulous orifices through which pus flowed easily under pressure (Fig. 1). External genitalia and other clinical examinations were normal. Laboratory blood tests were normal, except for an elevated CRP level of 52 mg/mL. Methicillin-resistant *Staphylococcus aureus* was isolated from the pus culture. A CT scan performed after a contrast injection showed a relatively well-limited mass with heterogeneous enhancement and central hypodensity related to the mesh (Fig. 2). En bloc removal of the mass and infected mesh was indicated. The procedure was performed under spinal anesthesia. The elements of the spermatic cord were intimately adherent to the mass, making the dissection laborious. The excision was completed with en bloc removal of the inflammatory mass (Fig. 3). Further pathological examination of the surgical specimen revealed an inflammatory granuloma without cellular atypia, compatible with a foreign body reaction. The inguinal wound was closed with a suction drain and left in place for 7 days. On the third post-operative day, a scrotal swelling appeared, and on the tenth day, there was an abundant flow of pus throughout the inguinal wound. Scrototomy allowed the diagnosis of ischemic necrosis of the left testicle. An orchiectomy was performed, and the patient received local care for the surgical wound until complete healing was achieved in 27 days. The surgical wound had healed completely one year post-operatively, indicating effective evolution. The examination revealed no signs of hernia recurrence.

## DISCUSSION

Infection is a relatively rare complication of inguinal hernial mesh repair [4]. The exact frequency is difficult to establish because diagnostic criteria vary between studies. It was less than 1% in several studies [1, 2, 5]. In addition to the properties of the mesh and the conditions of its implantation, the main risk factors identified are diabetes mellitus, smoking, and obesity [1]. These factors were not observed in our patient. Infection is more likely to be related to intraoperative contamination.

The diagnosis of a mesh infection is usually not problematic. Signs of mesh infection may appear within a variable timeframe [2]. Early infections, as in our case, are differentiated from late-onset infections, which can occur up to 11 years after prosthesis implantation [4]. In half of the cases, the infection is diagnosed within 1 month of surgery [2]. The diagnosis is essentially clinical, with obvious signs such as wound inflammation and pus discharge, as in our patient. Imaging, especially abdominal CT, can help diagnose late-onset infections [3]. In our case, where the diagnosis was obvious, it was more important to specify the extension of the infectious process to optimise the therapeutic indication. Mesh infections cause considerable morbidity and impair the patient’s quality of life. The goal of treatment is to eliminate the infection while avoiding recurrence. No consensus has been established regarding the appropriate treatment. Infection control is rarely possible without the complete removal of infected mesh [2, 5]. We observed that partial removal of the mesh was initially performed; however, controlling the infection was unsuccessful. Although complete removal provides the best chance of cure, it is not without complications. It sometimes causes iatrogenic lesions depending on the location of implantation [3]. In our case, it was probably an injury to the spermatic blood vessels, which resulted in necrosis of the testicle. Testicular ischemic lesions are well known to occur during inguinal hernia surgery, but progression to necrosis is exceptional [6]. In our case, extensive dissection coupled with chronic inflammation was probably responsible for venous lesions rather than arterial lesions because of abundant arterial supplies [7]. Extensive dissection and removal of the mesh also pose the problem of hernia recurrence. These recurrences are rare and allow simple monitoring after mesh removal [2]. As in our case, more than 80% of patients do not have recurrence because of fibrosis induced by inflammation [3–5].

In conclusion, the insertion of a mesh is a common procedure but by no means trivial. Infection is a serious complication that initiates a long and painful journey for patients. Although healing is achieved, significant sequelae may occur. The prevention of infection remains a constant preoccupation.

## CONFLICT OF INTEREST STATEMENT

The authors declare that the research was conducted in the absence of any commercial or financial relationships that could be a potential conflict of interest.

## FUNDING

The authors declare that they have no funding

## INFORMED CONSENT

Written informed consent was obtained from the patient for the publication of images and clinical data in this article.
